# p66Shc as a switch in bringing about contrasting responses in cell growth: implications on cell proliferation and apoptosis

**DOI:** 10.1186/s12943-015-0354-9

**Published:** 2015-04-08

**Authors:** Sahar S Bhat, Deepak Anand, Firdous A Khanday

**Affiliations:** Department Of Biotechnology, University of Kashmir, Srinagar, 190006 Kashmir India; Department of Life Sciences, King Fahad University of Petroleum and Minerals, Bld: 7, Room: 129, Dhahran, 31261 Kingdom of Saudi Arabia

**Keywords:** p66Shc, Cell proliferation, Cancer, ROS, Apoptosis, p53

## Abstract

p66Shc, a member of the ShcA (Src homologous- collagen homologue) adaptor protein family, is one of the three isoforms of this family along with p46Shc and p52Shc. p66Shc, a 66 kDa protein is different from the other isoforms of the ShcA family. p66Shc is the longest isoform of the ShcA family. p66Shc has an additional CH domain at the N-terminal, called the CH2 domain, which is not not present in the other isoforms. This CH2 domain contains a very crucial S36 residue which is phosphorylated in response to oxidative stress and plays a role in apoptosis. Whereas p52Shc and p46Shc are ubiquitously expressed, p66Shc shows constrained expression. This adaptor protein has been shown to be involved in mediating and executing the post effects of oxidative stress and increasing body of evidence is pinpointing to its role in carcinogenesis as well. It shows proto-oncogenic as well as pro-apoptotic properties. This multitasking protein is involved in regulating different networks of cell signaling. On one hand it shows an increased expression profile in different cancers, has a positive role in cell proliferation and migration, whereas on the other hand it promotes apoptosis under oxidative stress conditions by acting as a sensor of ROS (Reactive Oxygen Species). This paradoxical role of p66Shc could be attributed to its involvement in ROS production, as ROS is known to both induce cell proliferation as well as apoptosis. p66Shc by regulating intracellular ROS levels plays a crucial role in regulating longevity and cell senescence. These multi-faceted properties of p66Shc make it a perfect candidate protein for further studies in various cancers and aging related diseases. p66Shc can be targeted in terms of it being used as a possible therapeutic target in various diseases. This review focuses on p66Shc and highlights its role in promoting apoptosis via different cell signaling networks, its role in cell proliferation, along with its presence and role in different forms of cancers.

## Introduction

The Shc family of proteins consists four members ShcA, ShcB, ShcC and ShcD, of which the best characterized to date is ShcA [1,2]. ShcA, or simply Shc, was identified in 1992 as an adaptor protein which linked the activated EGFR (Epidermal Growth Factor receptor) to Ras and the MAP (Mitogen Activated Protein) kinase cascade [3,4]. Shc is expressed as three isoforms which have the molecular weight of 66, 52 and 46 kDa respectively. These isoforms are designated according to their molecular weight. All three of these isoforms are encoded by the same genetic locus, although the lower molecular weight isoforms have alternate start sites resulting in different amino-terminal sequences [5]. Expression of 52/p46Shc and p66Shc is controlled by different promoters [6,7]. All three isoforms of the Shc family consist of the N-terminal phosphotyrosine-binding domain (PTB), the middle collagen homology domain (CH1), and the C-terminal Src-homology domain (SH2). p52Shc and p66Shc have overlapping N-terminal sequences of 46 and 110 amino acids, respectively, which distinguishes these isoforms from p46. These two isoforms have the cytochrome c binding domain (CB), which is not present in p46Shc. p66Shc is the longest isoform of the three and also consists of the collagen homology domain (CH2), which is unique to it [8,6].

### Role of Shc isoforms in the propagation of mitogenic signals

p46Shc and p52Shc are ubiquitously expressed, while p66Shc is expressed at different level in various tissues. The CH1 domain of p46/p52Shc has three tyrosine residues which are phosphorylated in response to a number of cell surface ligand activated receptors. p52Shc binds to phosphorylated receptors via the PTB domain or the SH2 domains in response to growth factors, and is phosphorylated on these tyrosine residues (Y239/240, Y317) within the CH1 regions. Grb2 (Growth Factor Receptor-bound Protein 2) forms a complex with SOS (Son of Sevenless) protein. The Grb2/Sos complex is recruited by the phosphorylated p52/p46Shc proteins. This Grb2/Sos complex is then docked onto these phosphotyrosine residues, via direct interaction with the Grb2-SH2 domain, which ultimately leads to the activation of Ras proteins [1,7-10]. This coupling is implicated in the cytoplasmic propagation of mitogenic signals. p66Shc, upon stimulation by growth factors, is tyrosine phosphorylated by receptor tyrosine kinases. p66Shc upon tyrosine phosphorylation binds Grb2, and is unable to activate the Ras-MAPK-Fos pathways [6,11]. Ras signaling pathway is also inhibited when p66Shc is over-expressed upon stimulation by growth factors or cytokines [11-13]. Stimulation of MEK (MAPK-ERK Kinase)/ERK (Extracellular-signal Regulated Kinase) pathway by Insulin Growth Factor (IGF-1) is also inhibited by p66Shc. This pathway is required by the actin cytoskeleton phosphorylation in skeleton muscle myoblasts [14].

Another adaptor protein E3b1 together with Eps8 also plays a role in regulating the phosphorylation, reducing ubiquitilation and increasing the stability of p66Shc protein through Rac1 [15]. Sos1 can exist in a complex with Eps8/E3b1 and the complex of Sos1/Eps8/E3b1 leads to the activation of Rac1 [15]. p66Shc plays the role of a switch to dissociate Sos1 from the Grb2/Sos1 pool to Eps8/E3b1 pool. This in turn leads to Rac1 activation and as a result leads to an increase in the generation of oxidants [10-12,16]. Increased binding of p66Shc to activated EGFR and Grb2 occurs during severe oxidative stress. This binding leads to the dissociation of the Sos1 adaptor protein from the EGFR recruited signaling complex. These events lead to the termination of the Ras/MEK/ERK activation [17].

### Interplay of p66Shc and ROS – implications in aging and diseases

p66Shc is also a part of a signal transduction pathway that is activated by increased intracellular ROS, leading to apoptosis. The oxidative stress resistance and lifespan of a cell and an organism is increased by mutation of this pathway. The accumulation of oxidatively damaged macromolecules occurs in virtually all aging mammals [18]. In C. elegans and Drosophila, it has been seen that the genes controlling ROS metabolism play a role in determining lifespan. The decreased ROS production may be responsible for increased lifespan [19,20]. ROS and p66Shc have also been implicated in many diseases. Insulin has been seen to activate the redox enzyme activity of p66Shc specifically in adipocytes and p66Shc-generated ROS regulates insulin signaling. Deletion of p66Shc *in vivo* has been observed to increase metabolic rate as well as decrease fat mass and resistance to diet-induced obesity. p66Shc generated ROS regulates the effect of insulin and may lead to acceleration of aging by favoring fat deposition and as a result fat related disorders [21].

Furthermore, depletion of p66Shchas been shown to lead to Warburg effect, causing enhanced glycolysis and increased allocation of glucose-derived carbon into anabolic metabolism. This role of p66Shc has been observed in mice that are deficient in p66Shc. These p66Shc deficient mice show resistance to diabetes and obesity. This altered metabolism was seen to be mediated by mTOR (mammalian target of rapamycin). This indicates towards an inhibitory role of p66Shc in anabolic metabolism, unlike other isoforms of Shc [22]. Stimulation of p66Shc expression by hypercholesterolemia was observed in platelets. This lead to increased ROS levels in platelets in addition to hyperactivity and hyper aggregation in hypercholesterolemia. These effects were mitigated by down regulation of p66Shc [23]. p66Shc levels were also seen to increase progressively in failing myocytes that were affected by pacing-induced dilated cardiomyopathy. Pacing-induced dilated cardiomyopathy is characterized by an increased production of ROS and apoptosis. p66Shc, however, was undetectable in case of healthy cardiomyocytes [24]. p66Shc, therefore, plays a key role in cardiovascular diseases and obesity by regulating intracellular redox balance and oxidative stress levels [25].

**Figure 1:**
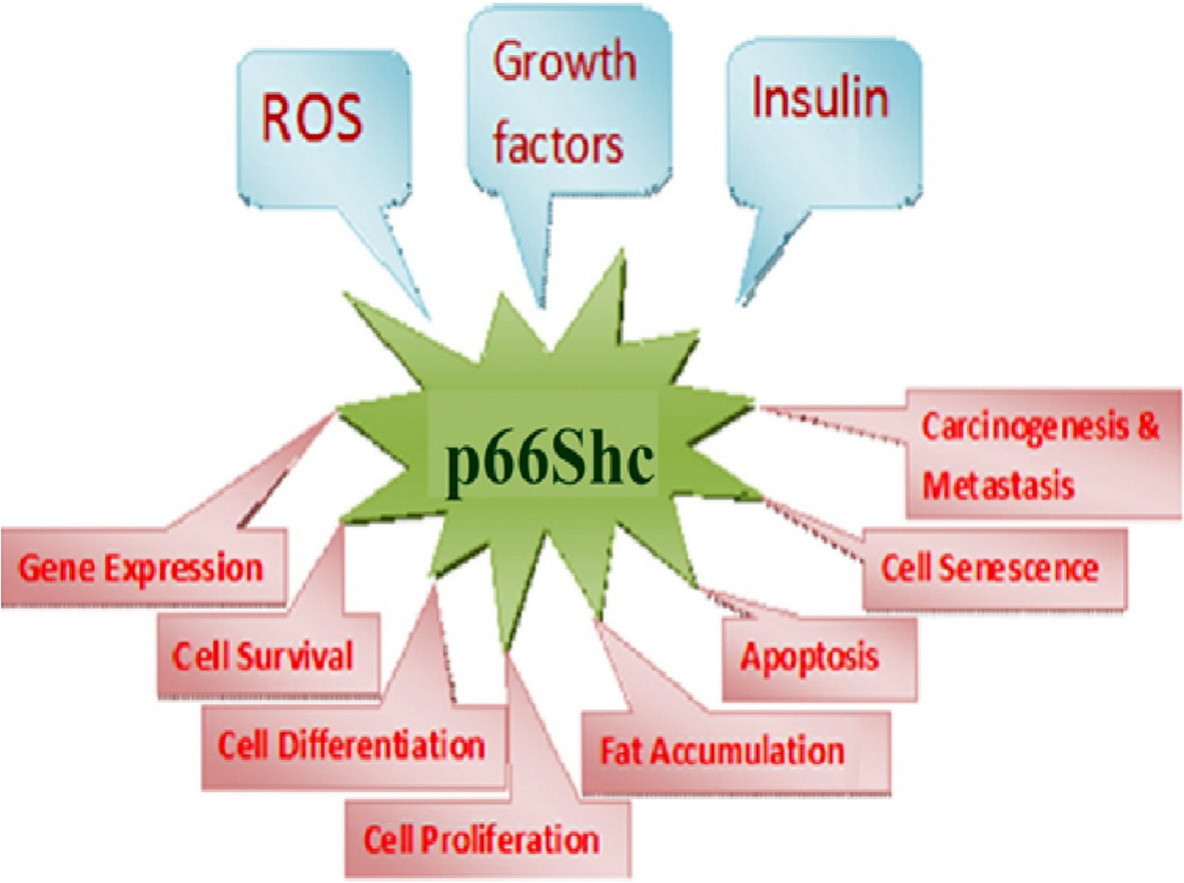
**Multi-faceted properties of p66Shc – different stimuli and different responses.**

p66Shc has also been shown to contribute to EAE (experimental autoimmune encephalomyelitis) induced neuronal damage. It does so, most likely, through the opening of PT pore that triggers mitochondrial swelling and leads to neurodegenerative stress [26]. In β-amyloid-mediated cell toxicity, MKK6-p66Shc form an important signaling cascade, wherein β-amyloid leads to apoptotic cell death via phosphorylation on S36 residue of p66Shc. Here the phosphorylation is carried out by MKK6 [27]. β-Amyloid plays a role in Alzheimer’s disease and causes the generation of ROS [28] (Figure 1).

### p66Shc phosphorylations – causes and consequences

p66Shc has a unique CH2 region at the NH2 terminal. This CH2 region is of around 110 amino acids and, like the CH1 domain, is rich in glycine and proline residues. The CH2 region contains the unique and all important serine phosphorylation (S36 and S54) sites [11]. p66Shc also has a cytochrome C binding (CB) region between the CH2-PTB domains. This is primarily involved in the regulation of oxidative stress in the mitochondria [29]. The CH2 domain, which is unique for p66Shc, may cause the selective regulation of translation of p66Shc protein. In addition to this, the utilization of two alternate promoters at the Shc locus may also play a role in the constrained expression of p66Shc, as opposed to the ubiquitous expression of p52Shc and p46Shc [30]. The expression of p66Shc is missing in peripheral blood lymphocytes, hematopoietic cell lines and neurons, its expression varies in breast and prostate cancer cell lines, whereas, it is primarily expressed in epithelial cells [5,11,31-34].

Under oxidative stress the S36 residue of p66Shc is phosphorylated. Treatment with an iron-containing porphyrin, hemin increased the phosphorylation of p66Shc at the S36 residue. In hemin treated K562 erythroleukemic cells p66Shc was transcriptionally activated through the ARE (Antioxidant response element)-Nrf2 (NF-E2-Related Factor 2) pathway [35]. In human colon carcinoma cell line RKO and in diploid human dermal fibroblasts suppression in the production of ROS was seen in shRNA-mediated knockdown of p66Shc and an increase in the production of ROS was observed upon overexpression of a recombinant p66Shc. These effects were not seen in the electron transport chain deficient ρ0-RKO cells. These ρ0-RKO cells are mitochondrial DNA depleted cells [36].

**Figure 2:**
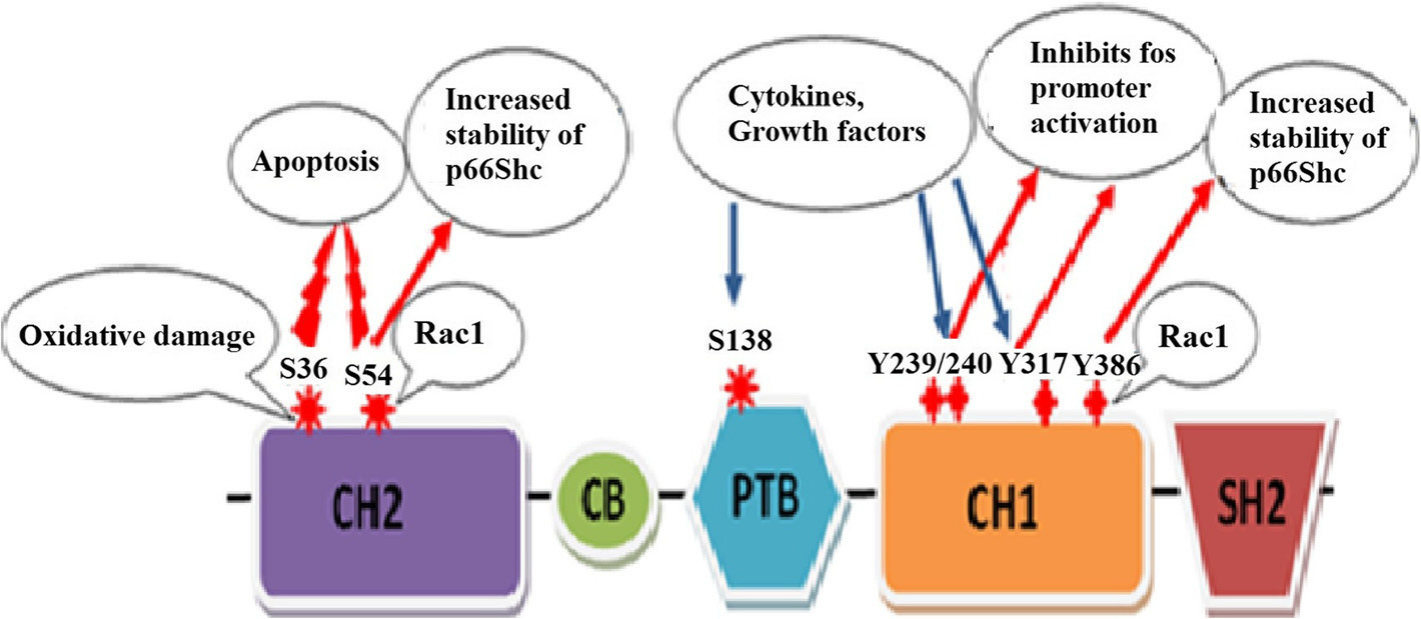
**Triggers and responses of phosphorylation of p66Shc residues.**

Till now most of the studies have demonstrated that p66Shc shows a pro-apoptotic and pro-oxidative role. Up to 30% extension in lifespan was seen in p66Shc knockdown mice. These knockdown mice also showed better handling of ROS and oxidative stress [6]. Mutagenesis of S36 to alanine results in a p66Shc variant that is unable to induce apoptosis. Therefore, S36 appears to be a critical regulatory site for the apoptotic activity and oxidative stress response of p66Shc [30,33,36-38]. This phosphorylation of the S36 residue of p66Shc in response to oxidative stress is done by PKCβ, which is activated by oxidative stress [39]. Oxidative stress gives a stimulus to mouse thymocytes, peripheral blood lymphocytes and splenic T-cells and these cells then attain the ability to express p66Shc [12]. Phosphorylation of p66Shc on the S36 residue has been shown to have a pro-apoptotic effect in different cell lines. The responses of S36 phosphorylation depends on the kinase(s) that mediate this process. The kinase(s) may differ (MAPK, stress-activate JNK and P38) depending on the cell type or the type of inducement [40-43]. When faced with oxidative stress conditions, S36 on p66Shc is phosphorylated which leads to the activation of p66Shc [44] (Figure 2). This activated p66Shc sensitizes to apoptotic stimuli after it is dissociated from an inhibitory complex [45]. Apoptotic death induced by oxidative stress is dependent on p53 and knockout of either p53 or p66Shc leads to resistance. This suggests that p66Shc is downstream of p53 in the pathway leading from ROS to apoptosis [46].

The S54 site in the CH2 domain of p66Shc is important for its stability [16]. The proteolytic PEST signal sequence of p66Shc is disguised by the phosphorylation of S54 in the CH2 domain and the T386 in the CH1 domain of p66Shc. These phophorylations are mediated by Rac1, and these Rac1 mediated phosphorylations lead to increase in the stability of p66Shc adaptor protein [16,47]. Conversely, it was also seen that p66Shc leads to the activation of Rac1 through the mediation of exchange factor Sos1 [10-12,16].

Recently it was seen that in SH-SY5Y cells or in the mice cortex, exogenous H_2_S inhibited the mitochondrial ROS production and as a result, phosphorylation of p66Shc and lead to sulfhydration of p66Shc on C59 residue [48]. ROS are known to regulate canonical Wnt signaling. The canonical Wnt ligand, Wnt3a also induces phosphorylation of p66Shc in endothelial cells. The knockdown of p66Shc inhibited both β-catenin dephosphorylation that is stimulated by Wnt3a as well as β-catenin dependent transcription. This inhibition was seen to be reversed by the overexpression of p66Shc, independent of Wnt3a. β-catenin dephosphorylation brought about by exogenous H_2_O_2_ was also seen to be mediated by p66Shc [49]. Many stimuli that are engaged in tyrosine phosphorylation of p66Shc also bring about the phosphorylation of S138 in the PTB domain [37]. The association of p66Shc adaptor protein in tyrosine phosphorylation signaling pathway is well known. Shc adaptor proteins, primarily p66Shc, transmit activated tyrosine phosphorylation signaling, which points to their possible role in growth regulation including carcinogenesis and metastasis [50].

### p66Shc and apoptosis

The increased levels of ROS act as the trigger to interfere with many cellular processes. These triggers result in inhibition of cell proliferation and induction of apoptosis. Apoptosis is the process of programmed cell death that takes place in multicellular organisms and comprises of many cellular events like cellular blebbing, nuclear fragmentation, chromosomal DNA fragmentation and ultimately cell death. p66Shc is known to have a role in apoptosis triggered by oxidative stress. The affirmation to this has been seen in a number of studies. Oxidative stress gives a stimulus to mouse thymocytes, peripheral blood lymphocytes and splenic T-cells and these cells then attain the ability to express p66Shc [40,45-48,50,51]. p66Shc overexpression lead to increased stress induced apoptosis [6]. p66Shc-/- cells are unable to restore pro-apoptotic responses when exposed to H_2_O_2_. All these studies point to the fact that p66Shc is needed for bringing about apoptosis. Phosphorylation of p66Shc on the S36 residue has been shown to have a pro-apoptotic effect in different cell lines. Upon expression of p66S36A mutant into these p66Shc-/- cells, they are still not able to restore the pro-apoptotic responses [6,28-30].

The gene encoding p66Shc has also attracted major interest in aging research in the recent years since it plays a vital role in apoptosis induced by oxidative stress. p66Shc is known to have a crucial role in the regulation of intracellular ROS levels [46]. The accumulation of oxidatively damaged macromolecules occurs in all aging mammals [18]. Activity of the mammalian forkhead homolog, FKHRL1, was seen to be increased and the redox dependent forkhead inactivation was seen to be reduced in p66Shc-/- cells. In addition to this, an increase in hydrogen peroxide scavenging as well as oxidative stress resistance was observed upon expression of FKHRL1. This points to a functional relation between forkhead protein, p66Shc and ROS, the three regulators of aging [52].

ROS can either originate from exogenous sources such as ultraviolet and ionizing radiations, or it can be endogenous (mitochondrial and certain enzymes). ROS has been implicated in the damage of cellular proteins, lipids and DNA. This oxidative cellular damage is apparently the main origin of aging. It causes mutations and deletions in both nuclear as well as mitochondrial DNA [18,44,53]. Various studies have revealed the association of cellular senescence and oxidative stress up-regulation of ROS in humans, with the increased expression of p66Shc [54-56]. The mitochondria contributes to the major portion of intracellular ROS production that is leaked from the electron transport chain [57]. p66Shc has emerged as a part of this mitochondrial ROS production. ROS production in mitochondria is regulated by many factors such as mitochondrial membrane potential, substrate supply and the level of metabolic activity. The mitochondrial pool of p66Shc forms a complex with Hsp70. Hsp70 is a chaperonin protein that is involved in protection from oxidative stress induced mitochondrial damage. When stimulated by UV radiation, this complex is released, pointing toward the role of Hsp70 in inhibiting the apoptogenic property of p66Shc [45]. In response to oxidative stress, part of the cytosolic pool of p66Shc is translocated to the mitochondria, which already has as high as 20 percent of fibroblast p66Shc localized in it [45]. p66Shc acts as an oxidoreductase in the mitochondria. After binding to cytochrome C, p66Shc shuttles electrons from cytochrome C to molecular oxygen [29]. This redox activity of p66Shc leads to the increase in ROS production caused by the recombinant expression of p66Shc. This explains the decrease in ROS levels typical of p66Shc knockout cells [58]. MEFs (Mouse embryo fibroblasts) from p66Shc-/- transgenics were found to be resistant to apoptotic death that is induced by oxidative stress. Furthermore, it has been demonstrated that the cells derived from p66Shc-/- mice show a decrease in the oxidation of the C8 of guanine, as well as, in the mutation of mitochondrial DNA [59].

The p66Shc-/- fibroblasts cells have altered bio-energetic properties vis-à-vis decreased resting and maximal oxidative capacity. This suggests that deletion of p66Shc may extend lifespan by shifting metabolic energy away from oxidative and toward glycolytic pathway [60]. One can, therefore, interpret that p66Shc mediates triggering of apoptosis [61], and its deletion leads to a significant increase in lifespan. It is apparent that the main reason for the prolonged lifespan of p66Shc-/- mice is the decreased intracellular ROS levels. The survival and reproduction of p66Shc-/- mice in a population living in an outdoor enclosure for a year was studied. It was observed that p66Shc-/- mice had defects in fat accumulation, thermoregulation as well as reproduction, pointing to the role of p66Shc in energy metabolism and to the difference in the results in protected laboratory conditions and natural conditions [62].

### p66Shc and crosstalk with p53

**Figure 3:**
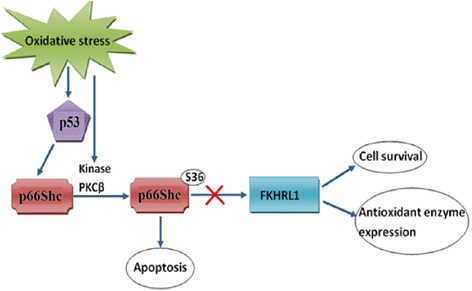
**Schematic representation of the possible role of p53 in the p66Shc mediated oxidative stress induced apoptosis.**

The tumor suppressor p53 is critically involved in oxidative stress-dependent apoptosis. p53 has a role in mediating the oxidative stress response of p66Shc, since p53 is up-regulated upon treatment with H_2_O_2_ and UV. p53-/- MEFs also show resistance to UV-induced and H_2_O_2_-induced apoptosis [6]. It has been demonstrated that p66Shc serves as a downstream effector of p53 [46]. Increased expression of p53 in DLD-1 colon cancer cells and WT MEFs, lead to a significant increase of p66Shc [46]. p66Shc is not involved in functions of p53 such as cell cycle arrest but p66Shc does regulate the p53-dependent apoptosis [46] (Figure 3). Activation of p53 leads to an increase in the ROS levels via the release of cytochrome C from the mitochondria and the apoptosome assembly. p66Shc-/- cells failed to increase the ROS levels upon increasing the expression of p53 because they were unable to trigger cytochrome C release. The levels of ROS were increased in these after the expression of p66Shc in p66Shc-/- MEFs, pointing to the crucial role of p66Shc and p53 in the regulation of intracellular ROS levels [46].

### p66Shc and regulation of anoikis

The most important property of multicellularity is that cells can grow and differentiate only when in the correct context within a tissue, and can remove themselves by apoptosis when they are not. Specific interactions with the ECM (extracellular matrix) help the cells to sense their location as well as that of the neighboring cells. When these cell-ECM interactions are incorrect, apoptosis takes place. This kind of location specific apoptosis is termed anoikis. Anoikis is a special case of programmed cell death that is initiated by cell detachment instead of the normal pro-apoptotic signals [63]. Anoikis is an essential mechanism for making sure that cells maintain their proper positioning and avoid colonization of distant organs. Aniokis resistance is a vital step of cancer progression and metastatic colonization. Cancer cells develop anoikis resistance by a variety of mechanisms such as the over activation of receptors, modulating matrix stiffness, increasing oxidative stress etc. [64].

Cells that lack p66Shc have been shown to escape anoikis. Anoikis in these cells was restored with the reexpression of p66Shc. p66Shc restores anoikis via RhoA activation. Transient as well as stable transfections with p66Shc lead to cell death in detached cells, but not in adherent cells. In contrast to apoptosis caused by oxidative stress, this ability of p66Shc to confer anoikis properties is not dependent on S36 phosphorylation of p66Shc [65]. In addition to this, it was also seen that in the absence of p66Shc native K-Ras behaves like oncogenic Ras. Ras was activated in the absence of p66Shc in normal as well as malignant cells. This hyperactivated Ras lead to the inhibition of anoikis rather than leading to increased proliferation rates. The metastatic effect in these cells was lost upon re-expression of p66Shc [66]. Thus p66Shc functions as a regulator of anoikis and has a role in the prevention of metastasis.

### p66Shc and cell proliferation

Cell proliferation, migration and adhesion are the main properties of a tumor cell. Tumor cells proliferate, migrate and adhere to target tissue. These steps allow the tumor cell to obtain metastatic phenotype. Cell proliferation and migration depend on the signals transmitted by growth factors and adhesion proteins [67]. These processes are mainly regulated by the Rho family proteins and ERK cascades [19,67-69]. Shc binding has been shown to be needed in cell motility induced by the nerve growth factor receptor trkA [70]. The processes of migration, proliferation and adhesion require the rearrangement of actin cytoskeleton. It involves the release of pre-existing cell-matrix contacts and formation of new integrin substratum contacts [71]. p66Shc mediates cell proliferation mediated by growth factor receptor signaling, and also plays a crucial role in cell migration and adhesion [72,73].

p66Shc also plays a crucial role in proliferation of epithelial cells. The processes of cell proliferation and apoptosis have to be well balanced. The balance of these two processes is governed by signal transduction machinery [74]. Any imbalance between these two processes can cause various life threatening diseases like autoimmune diseases, cancer etc. [75-77]. The Shc proteins are one of the most extensively studied adaptor proteins for understanding the role of adaptor proteins in cell signaling pathways. As a result the role of Shc proteins in various signaling pathways has received much attention [11,30,78-80]. Steroid hormones play a role in regulating the expression of the Shc proteins [50]. Steroid hormones also play a role in various cancers and regulate processes like cancer cell proliferation and progression [81]. The excess of steroids in hormone related cancers causes aberrant growth regulation due to elevated levels of various growth factors [82-84]. In addition to this, well established connections between steroid hormones and tyrosine phosphorylation signaling are already present [70,82,84-89]. Carcinogenesis is, in turn, mainly regulated by growth factors and their receptors. These regulate processes like cell proliferation, motility, migration, invasion etc. [4,5,46,70,90-95]. It is quite evident that Shc proteins play a role in carcinogenesis. Aberrant expression of p66Shc has been seen to be involved in carcinogenesis [8,31,79,80,96-98].

### p66Shc in breast cancer

ARF1 has been found in complex with Grb2 and p66Shc upon EGF stimulation of the basal like breast cancer MDA-MB-231 cell line. The small GTPases ARF1 and ARF6 have been shown to be activated downstream of the EGFR and act as key regulator of growth, migration and invasion of breast cancer cells [99]. Estrogen treated breast cancer MCF-7 cells also showed an increase in both p66Shc protein level as well as the cell growth [79]. The metastatic variant of breast cancer MDA-MB-231 cell line F-11 cells have a 3-fold higher level of p66Shc expression as compared to MDA-MB-231 cells, whereas the levels of p46 and p52 isoforms remained unchanged [31]. The levels of p66Shc were measured in primary breast cancer specimens. The breast cancer specimens that were associated with lymph node metastases, showed an increase in the level of p66Shc. Higher levels of p66Shc correlate with higher metastatic potential in breast cancer [31]. Decrease in ShcA levels or conversely the expression of a dominant-negative ShcA mutant lead to the blocking of motility induced by TGF-β and furthermore also leads to the inhibition of invasion of Neu/ErbB2- expressing breast cancer cells [13]. p66Shc forms a trimeric complex with alpha-1-syntrophin and Grb2. The formation of this complex triggers cell proliferation and migration of MCF-7 and HBL-100 breast cancer cell lines. The role of p66Shc in cell proliferation was analyzed in these cells by knock-down approach, where siRNA against p66Shc was used. A marked decrease in cell proliferation was observed by this method, pointing to the role and importance of p66Shc in cell proliferation in breast cancer cell lines [100].

### p66Shc in prostate cancer

p66Shc protein levels were seen to be closely correlated to the growth rate of prostate cancer cells. p66Shc protein levels were also seen to be significantly higher in prostate cancer adenocarcinomas as compared to the adjacent benign glandular cells [79,97]. p66Shc protein level was seen to be 4–10 times higher in rapidly growing prostate cancer cells such as PC-3 and DU145 cells, as compared to slow growing cells such as LNCaP C-33 [101]. The p66Shcprotein level as well as the cell proliferation rate is increased in these slow growing human prostate cancer cells when they are treated with androgen or EGF. These levels are higher than the corresponding cells grown without the stimulus [79]. The role played by p66Shc protein in regulating the growth of prostate cancer cells has been supported by both cDNA as well as siRNA approaches [101]. Cell proliferation was observed when the expression of p66Shc was increased by cDNA transfections. Knock-down of p66Shc by its siRNA decreased cell growth rate. Decreased Ser36 phosphorylation leads to decreased apoptosis since phosphorylation of Ser36 of p66Shc serves as a sensor of apoptotic pathway [7]. The elevated level of p66Shc hence, plays a critical role in up-regulating androgen-regulated prostate cancer cell proliferation and contributes to the tumorigenicity of these cells. Furthermore, LNCaP C-81 and PC-3 prostate cancer cells which have high levels of p66Shc, exhibit higher metastatic potential than LNCaP C-33 cells [101]. In androgen stimulated prostate cancer cells elevated p66Shc expression correlated with increased ROS levels and increased cell proliferation. This increase in cell proliferation was blocked by treating with antioxidants as well as by the knockout of p66Shc. Androgens lead to the increased expression of p66Shc, which in turn leads to an increased production of ROS [102].

### p66Shc in esophageal cancer

**Figure 4:**
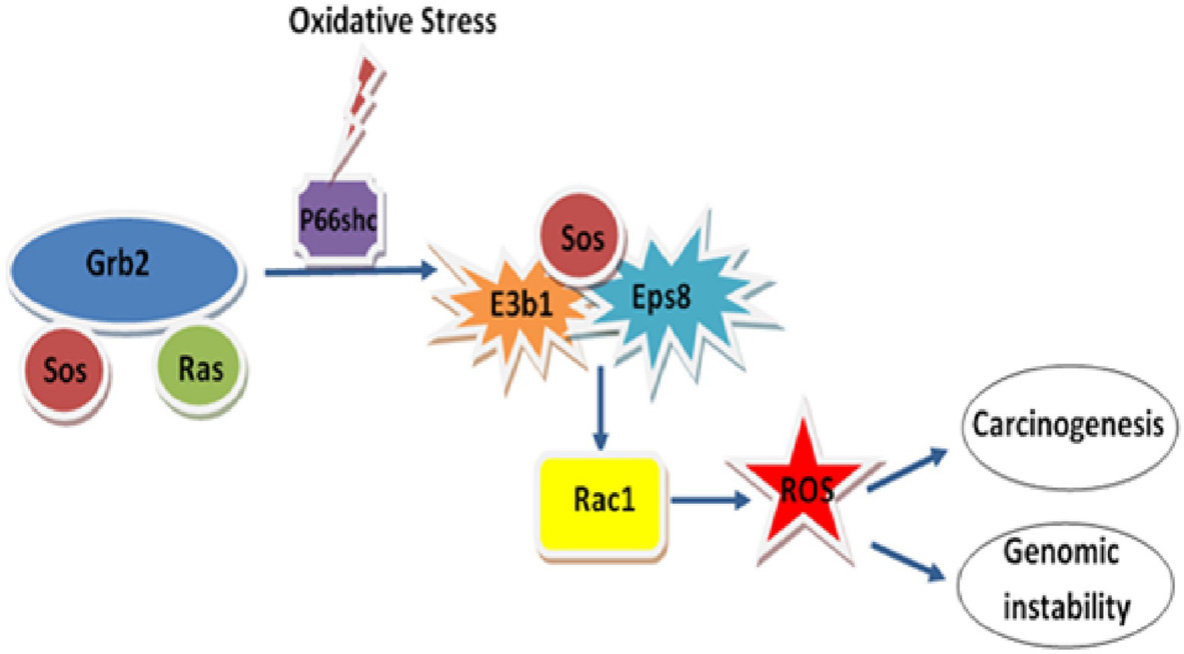
**Schematic representation of the role of EpS8, E3b1 and Rac1 in oxidative stress induced p66Shc mediated carcinogenesis.**

A consistent increase in the p66Shc protein levels were observed in ESCC (esophageal squamous cell carcinoma) and EAC (esophageal adenocarcinoma). The controls of ESCC showed a lower basal level of p66Shc expression [75]. In the same study a 4–8 fold increase in the level of expression of p66Shc was observed in ESCC and a 2–3 fold increase was seen in EAC by densitometric analysis. The pronounced activity of various gastric juices, bile acids and digestive enzymes in lower gastrointestinal tract may be responsible for the higher levels of ROS in esophageal adenocarcinoma [103], which can be a reason for the higher basal expression level of p66Shc in EAC as compared to the lower levels in ESCC [75]. The levels of downstream targets of p66Shc were also checked and higher levels of EpS8 and Rac1 were observed in ESCC, as well as, EAC as compared to adjacent normal tissue [75]. p66Shc promotes a trimetric complex formation between EpS8, E3b1 and Sos1, which in turn leads to increased Rac1 activity [31] (Figure 4).

### p66Shc in thyroid cancer

The expression of p66Shc was seen to be increased in all kinds of proliferating thyroid tissues, but not in the normal thyroid tissue of the same patient [104]. This data makes sense in the view of the fact that rapid growing cells have activated metabolic reactions and those lead to increased ROS production [105]. p66Shc is involved in the oxidative stress signaling cascade which leads to the production of H_2_O_2_ [6], through the p66Shc-Rac1 pathway [47].

### p66Shc in lung cancer

Aiolos, which is often expressed in lung cancers and correlated with reduced patient survival, has been seen to be inversely correlated with the expression of p66Shc in lung cancer tissue as well as in single cells [106]. On the other hand, Shc has been known to amplify the mitogenic signal delivered by the hepatocyte growth factor receptor Met in BN-14 mouse bile duct as well as A549 lung carcinoma cells [30]. p66Shc deficiency in human lung adenocarcinoma A549 cells was seen to mitigate the low nutrient induced autophagy. p66Shc inhibition in these cells by shRNA also lead to prolonged phosphorylation of (ERK)1/2 kinase. Cleavage of caspase 7, but not of caspase 6 and 9 was also observed under these conditions in the A549 cells, pointing to the fact that p66Shc may play a role in regulating autophagy and apoptotic resistance under conditions of limited nutrients [107]. In clinical human lung cancer samples and cancer cell lines, methylation of a specific CpG site in the post-transcriptional region was seen to be correlated with the p66Shc repression. The association of stress related transcription factor Nrf2 requires the demethylation of the Nrf2 binding site in the promoter of p66Shc, in order for Nrf2 to promote the transcription of p66Shc. Nrf2 upregulation along with increased cell survival and tumor progression in these cancer cells might be as a result of the epigenetic repression of p66Shc [108].

### p66Shc in ovarian cancer

In ovarian cancer cells the level of ErbB-2 expression positively correlates with the level of p66Shc. ErbB-2 is a prognostic marker for ovarian carcinoma [34]. In ovarian cancer tissues, cancerous cells show higher levels of p66Shc proteins as compared to the adjacent non-cancerous cells [109]. In steroid treated ovarian cancer cells, p66Shc levels were up regulated, along with increased cell proliferation. When treated with the respective antagonists, these effects on p66Shc levels were competed out [50]. Treatment of ovarian cancer cells with proteasomal inhibitors lead to higher levels of p66Shc protein. Immunoprecipitation experiments have shown that the elevation of p66Shc levels by the treatment of steroids and proteasomal inhibitors was a result of reduced ubiquitination of p66Shc protein in these cells [110]. In a recent study, SHetA2 was seen to inhibit the binding of mortalin (HSPA9) binding to p53 and p66Shc in ovarian cancer cells [111]. SHetA2 has been identified of having cancer prevention properties and hence therapeutic activity. The role of p66Shc was studied with respect to regulating cell proliferation in ovarian cancer. It was observed that the slowest growing cell line, OVCAR-3, showed the lowest level of p66Shc expression. In addition to this, transient transfection with p66Shc cDNA expression vector in OVCAR-3 cell line resulted in an increase in cell proliferation [109].

### p66Shc in colon cancer

In colon cancer tissues, cancerous cells show higher levels of p66Shc proteins as compared to the adjacent non-cancerous cells [96]. p66Shc expression in benign, premalignant, and malignant gastric and colorectal lesions was investigated in a separate study. Normal colorectal and gastric tissue samples showed low or no p66Shc expression. As opposed to the normal tissue, the gastric and colorectal tumors showed moderate to high p66Shc expression [112]. It was also seen that colon carcinoma cell line RKO was more resistant to oxidative stress induced by hydrogen peroxide exposure or serum starvation when p66Shc was knocked out. Mitochondrial fragmentation was also reduced in these p66Shc deficient RKO cells. Mitochondrial fragmentation is in turn proportional to the amount of mitochondrial ROS, therefore, showing the role of p66Shc in the mitochondrial accumulation of ROS [113].

## Conclusion and prospective

**Figure 5:**
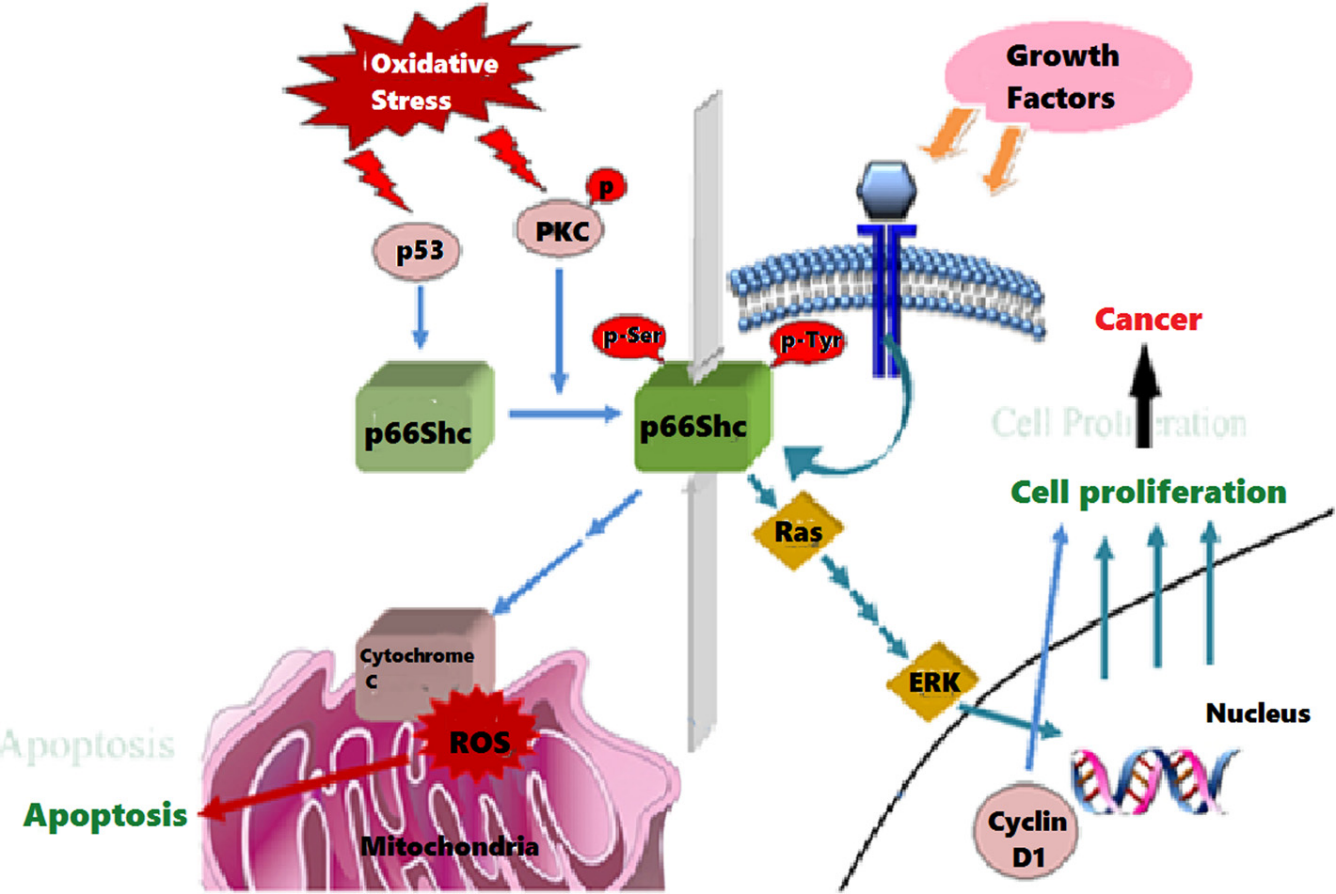
**p66Shc: A dual player – role of p66Shc in cell proliferation and cell proliferation.**

A recent study has shown the regulatory role of p66Shc-derived mitochondrial ROS. The p66Shc-derived ROS has a regulatory role on growth factor signaling by induction of protein tyrosine phosphatase (PTP) oxidation. It was seen that p66Shcdeletion and down regulation lead to decrease in PDGF-induced signaling and migration, by decreasing PDGF-induced PTP oxidation [114]. In summation, all these observations clearly show the role of p66Shc in cell proliferation and the presence of increased p66Shc expression in different cancer cell lines/tissues and its effects leading to higher metastatic ability in these cell lines. In addition to its role in cell proliferation, p66Shc is known for being an important apoptosis regulator. These observations point towards a dual, albeit paradoxical role of p66Shc. While p66Shc acts as a proto-oncogene on one side, showing increased p66Shc expression in different cancer cell lines/cancer tissues, and leading to increased cell proliferation and metastatic potential by targeting various signaling pathways. On the other hand it acts as a pro-apoptotic protein. Its absence in cells prevents or inhibits apoptosis and in addition to that it has been found to be a downstream effector of p53. p53 is a well-known tumor suppressor as well as a pro-apoptotic protein. In response to oxidative stress, the level of p66Shc activation increases and p66Shc−/− cells are unable to undergo apoptosis in response to oxidative stress. These properties of p66Shc belong to two different ends of spectrum, where its apparent role as a pro-apoptotic protein totally conflicts with its role as a proto-oncogene. p66Shc is not a unique protein falling in the category of dual regulators of cell growth. It is possible that p66Shc through growth factor stimulation/regulation may contribute to cell proliferation while as its regulation through oxidative stress might predominantly contribute to apoptosis (Figure 5). Varying levels of ROS may play a deciding role in contributing to these contrasting responses in the cell, where the intracellular levels of ROS may predominantly determine the route a cell will take. Since p66Shc is involved in the production and control of ROS, therefore, ROS seems to be an important factor in the cell vis-à-vis deciding the different cell responses. Steady state level of ROS or acute production of intracellular ROS may lead a cell towards different paths and ultimately different fates. The role of ROS levels in apoptosis is well established. A correlation between p66Shc expression, ROS levels and growth stimulation has been observed in prostate cancer cells where increased p66Shc levels as well as increased ROS levels were observed in addition to increased growth. This growth stimulation was abolished when the cells were treated with antioxidants [101]. It may be that, under normal ROS concentration, the cell will function normally. A slight increase in ROS level may lead to ROS acting as a secondary messenger at an elevated rate leading to increased or excessive cell proliferation. A higher outburst of ROS may contribute to oxidation damaged DNA in a cell and in turn might trigger the oxidation stress induced pathway via p66Shcthat leads to apoptosis.

p66Shc being a candidate protein that controls both cell death as well as cell proliferation and plays a crucial role in regulating longevity and cell senescence, by regulating intracellular ROS levels. These counteracting properties of p66Shc make it a perfect candidate for further studies in various cancers and aging related diseases. p66Shc can be targeted in terms of it being used as a possible therapeutic target in various diseases, by figuring out how to use p66Shc protein for balancing the ROS levels in the cells and tipping the scales in our favor.
